# An Exploratory Statistical Shape Modeling Analysis of Three‐Dimensional Foot Alignment in Female Patients With Progressive Collapsing Foot Deformity

**DOI:** 10.1002/jfa2.70127

**Published:** 2026-02-24

**Authors:** Takuma Miyamoto, Rich J. Lisonbee, Kassidy Knutson, Hiroaki Kurokawa, Akira Taniguchi, Yasuhito Tanaka, Amy L. Lenz

**Affiliations:** ^1^ Department of Orthopaedics University of Utah Salt Lake City Utah USA; ^2^ Department of Orthopedic Surgery Nara Medical University Kashihara Nara Japan; ^3^ Department of Mechanical Engineering University of Utah Salt Lake City Utah USA

**Keywords:** 3D foot alignment, flatfoot, progressive collapsing foot deformity, statistical shape model

## Abstract

**Introduction:**

Progressive collapsing foot deformity (PCFD) is a complex condition characterized by multiple combined deformities, with a higher reported prevalence in women. Most studies of PCFD morphology have focused on single joints and evaluated both sexes together, leaving the interrelationship of multiple joints in females specifically unassessed. In this study, we developed a multi‐domain statistical shape model (SSM) from reconstructions of simulated weight‐bearing computed tomography (SW‐CT) image data to investigate variations in 3D foot alignment in women by comparing the morphology of patients with PCFD to that of their asymptomatic control counterparts.

**Methods:**

We developed an SSM to analyze the 3D alignment in the foot, including the distal tibia, distal fibula, talus, calcaneus, navicular, cuboid, and each of the cuneiforms and metatarsal bones. Model results were compared between 23 female patients with PCFD and 23 female asymptomatic individuals.

**Results:**

Although the Chopart, subtalar, and Lisfranc joints were dorsiflexed and abducted, the Chopart and subtalar joints were inverted whereas the Lisfranc joint was everted, indicating that deformity of the Lisfranc joint occurred in the opposite direction to that of the subtalar and Chopart joints.

**Conclusions:**

The application of a multi‐domain SSM from SW‐CT to evaluate alignment deformities in patients with PCFD represents a new and novel approach to understanding the pathophysiology of this condition. In PCFD, the subtalar and Chopart joints showed coordinated changes, whereas the Lisfranc joint demonstrated variation in the opposite direction. Also, the talonavicular joint spreads out medially in a fan‐like direction in PCFD.

## 1 Introduction

Progressive collapsing foot deformity (PCFD) is a common issue with the foot and the ankle [1] characterized by clinical presentation of combined deformities thought to be driven by the collapse of the medial longitudinal arch [2, 3]. Although the primary pathogenic factor is thought to be posterior tibia tendon (PTT) dysfunction from early literature and clinical perspectives, the pathophysiology is not yet fully understood and PCFD may affect the subtalar (ST) joint, the Chopart joint, and the Lisfranc joint [4]. Therefore, PCFD deformities are likely to be affected by polyarticular relationships with many bony degrees of freedom; however, most studies to date have evaluated single joints alone, and the interrelationship of multiple joints has not yet been assessed. In addition, PCFD occurs three times more often in women [5], and previous studies have reported sex‐related differences in hindfoot bone morphology, including a larger distal tibia in males and a longer and thinner calcaneus in females [6]. However, most studies of PCFD morphology have evaluated both sexes together.

In recent years, computed tomography (CT) has become a common tool in diagnosing and evaluating PCFD [7, 8]. While CT provides three‐dimensional (3D) information, most often in clinical practice, these images are evaluated using the same or similar two‐dimensional (2D) measurements used for plain film x‐rays [9]. Statistical shape modeling (SSM) provides a 3D evaluation of population‐based anatomical variation as well as groupwise shape differences of anatomies reconstructed from volumetric imaging data [9, 10, 11, 12]. Recent advancements in SSM have made it possible to evaluate multiple domains or bones at once, providing the ability to study the variation in the alignment of a human joint [13].

The human foot adapts to different surfaces through compensatory motion among joints, and such compensatory patterns have also been reported in deformities like ankle osteoarthritis [14]. Although no similar studies have been conducted on PCFD, it is a whole‐foot deformity, and different joints may be either primarily affected by the deformity or exhibit compensatory changes.

In this study, we developed a multi‐domain SSM from reconstructions of simulated weight‐bearing computed tomography (SW‐CT) image data to investigate variations in 3D foot alignment in women by comparing the morphology of patients with PCFD to that of their asymptomatic control counterparts. It was hypothesized that the subtalar and Chopart joints show coordinated changes in PCFD, whereas the Lisfranc joint demonstrates a compensatory relationship in the frontal plane.

## 2 Materials and Methods

### 2.1 Study Design

**FIGURE 1:**
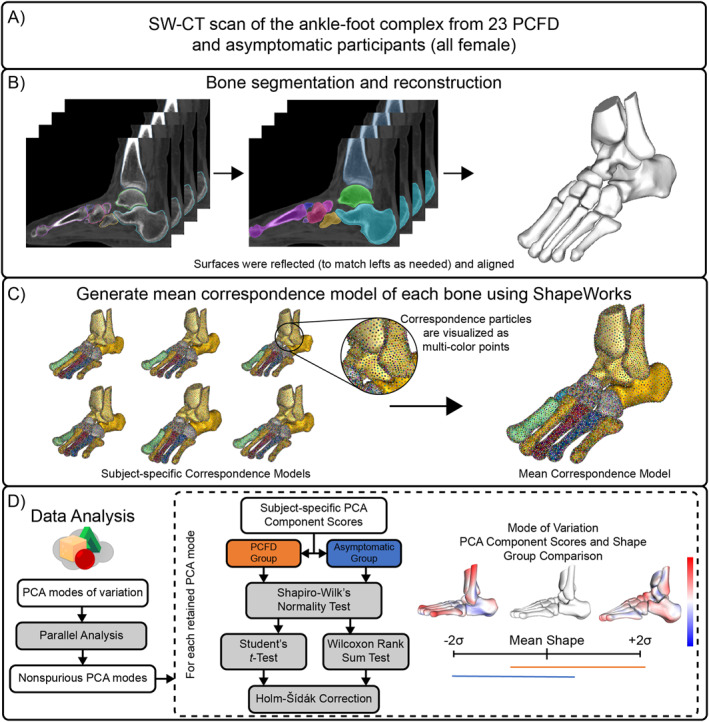
Computational workflow for this study: (A) SW‐CT scans of 23 individuals with PCFD and 23 asymptomatic participants (all female). (B) Within each SW‐CT scan, the tibia, fibula, talus, calcaneus, navicular, cuboid, all cuneiforms, and each metatarsal was semiautomatically segmented followed by verification and clean up, and these were consistently meshed to create 3D bone reconstructions. (C) A statistical shape model of each bone was developed from the 3D reconstructions to determine PCA modes of variation. (D) For each bone correspondence model, the nonspurious PCA modes were identified using a parallel analysis followed by group comparisons of shape and PCA component scores.

This is a retrospective comparative study. This study was approved by the institutional review boards of our affiliated institutions (No. 2872). An opt‐out statement regarding the application of medical data was published on our institute's website. This study was performed under the principles of the World Medical Association Declarations of Helsinki. The workflow of this study is presented in Figure 1.

### 2.2 Data Collection

A study was conducted on 35 female patients who underwent surgical treatment for PCFD at Nara Medical University from 2014 to 2019. The exclusion criteria included previous surgeries on the same limb, severe hindfoot and midfoot bone collapse, and unavailability of imaging data for the hind‐, mid‐, or forefoot bones selected for the study. Eventually, a sample of 23 patients presenting with PCFD was chosen (age: 65.1 ± 7.1 years, height: 154 ± 4.9 cm, weight: 60.4 ± 9.8 kg, and BMI: 25.1 ± 4.3 kg/m^2^). Patients were classified according to the PCFD classification system, which incorporates both clinical examination findings and radiographic parameters as described by Myerson et al. [4]. The PCFD classification [4] categorized one patient as 1B, one patient as 1C, two patients as 2B, six patients as 2C, 10 patients as 2D, and three patients as 2E. The asymptomatic control group consisted of 23 women with no history of ankle joint injuries, deformities, or surgical trauma (age: 46.7 ± 19.2 years, height: 155 ± 7.2 cm, weight: 58.2 ± 14.1 kg, and BMI: 23.9 ± 5.5 kg/m^2^).

For the study, a simulated weight‐bearing computed tomography (SW‐CT) was performed using an OPTIMA 660 device (General Electric, Boston, USA) with a 0.625‐mm slice thickness and a 512/512 matrix resolution. This was combined with DynaWell L‐Spine (Dyna‐Well Inc., Las Vegas, USA) to simulate a weight load of 300 N at the ankle neutral position [15]. Semiautomatic measurements of the Meary's angle, calcaneal pitch, and hindfoot alignment angle were taken from the SW‐CT images (DISIOR Bonelogic Ortho Foot and Ankle 2.1. Helsinki, Finland).

### 2.3 Bone Segmentation

For each participant, SW‐CT images were auto‐segmented to create 3D models of distal tibia, distal fibula, talus, calcaneus, navicular, cuboid, all cuneiforms, and all metatarsal bones (DISIOR 2.1, Bonelogic). DISIOR software was used to obtain standardized surface‐based reconstructions from CT data; however, this does not represent a full voxel‐based segmentation. These initial segmentations were then manually adjusted to finalize the surface reconstructions (Mimics 22.0, Materialise). Mimics was used because the semiautomated segmentation in DISIOR may produce minor inaccuracies, such as boundary misidentification or surface artifacts. Therefore, all segmentations were manually checked and refined to ensure accuracy. The generated bone surfaces were meshed and smoothed consistently using 3‐matics (3‐matic 16.0, Materialise) [7]. Preprocessing of the 3D bone reconstructions included mirroring right bones to represent left bones, aligning and centering using an iterative closest point algorithm [16] (Figure 1). Mirroring was performed to increase the effective sample size and to minimize the influence of left–right variability, consistent with prior shape modeling studies.

### 2.4 Statistical Shape Model

A multi‐domain SSM was performed with all 14 bones across 46 feet from 46 participants (23 PCFD patients and 23 controls) to generate statistical shapes using ShapeWorks (6.4.2, University of Utah) [11]. The methods used by ShapeWorks rely on particle‐based shape models to place landmarks on the shapes, using an optimization scheme [17]. The total particle count for each bone was as follows: 1024 for the distal tibia, talus, and calcaneus and 512 for distal fibula, navicular, cuboid, each of the cuneiforms, and metatarsals. These correspondence particle locations were analyzed to define mean shapes and quantify bone shape differences across the population. To assess the quality of particle placement, we used ShapeWorks evaluation tests (generality, specificity, and compactness) and adjusted the number of particles until stable results were obtained. This strategy reduces reliance on specific anatomical landmarks and minimizes bias, while capturing overall morphological variation in a data‐driven manner. A generalized Procrustes analysis was used to remove scale from the shape model analysis [17]. Correspondence particles from the SSM were evaluated via principal component analysis (PCA) based on a covariance matrix [11, 18, 19].

### 2.5 Statistical Analysis

PCA modes to be retained for analysis were determined using parallel analysis [20]. Within the retained PCA modes, PCA component scores within each retained mode were tested for normality using a Shapiro–Wilk test and then compared between groups using the appropriate two‐sample *t*‐test (parametric) or Wilcoxon rank sum test (nonparametric). PCA component score tests across modes for a specific domain were corrected using a Holm–Sidak correction to reduce the probability of type 1 error. For all statistical measures and comparisons, an alpha value of 0.05 was used (*p* < 0.05). These statistical analyses were performed in MATLAB. For each statistically significant determined mode, the distance between the mean surface and the ± 2 standard deviation (SD) shapes were calculated and visualized using CloudCompare (v2.11. alpha, www.cloudcompare.org). Modes of variation were described clinically based on the expertise of a foot and ankle orthopedic surgeon. In this paper, morphological orientation in the sagittal plane is defined as plantarflexion/dorsiflexion; motion in the coronal plane as inversion/eversion; motion in the transverse plane of the ankle, subtalar, and Chopart joint as internal/external rotation; and motion in the transverse plane of Lisfranc joints as adduction/abduction.

## 3 Results

**FIGURE 2:**
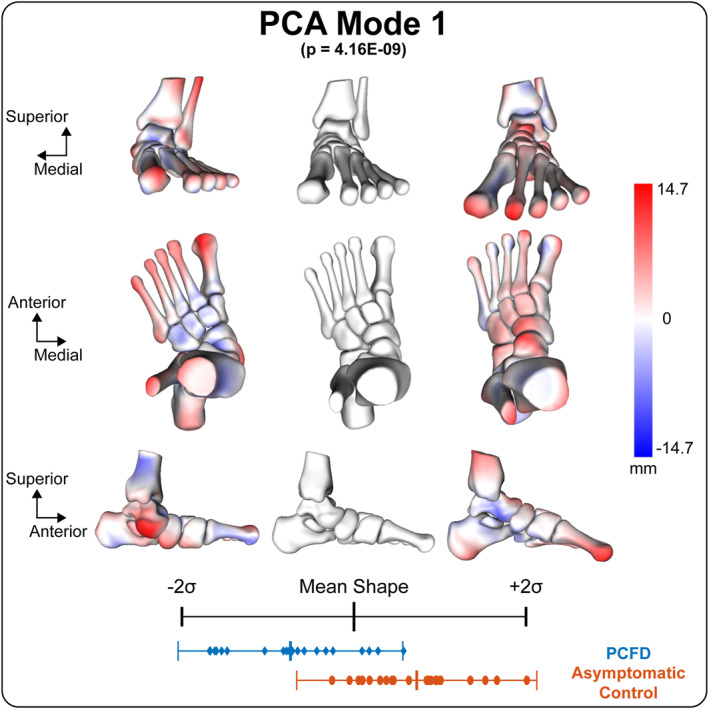
Multi‐domain PCA mode 1 quantitative visual description from the anterior, superior, and medial viewing perspectives, which describes 53% of the explained variance. The group PCA component score distributions for this mode of variation indicate statistically significant differences between PCFD and controls (*p* = 4.16 × 10^−9^). The red and blue colors represent positive and negative surface displacement, respectively, relative to the mean shape. Distances are calculated along the surface normal direction at each correspondence point.

**FIGURE 3:**
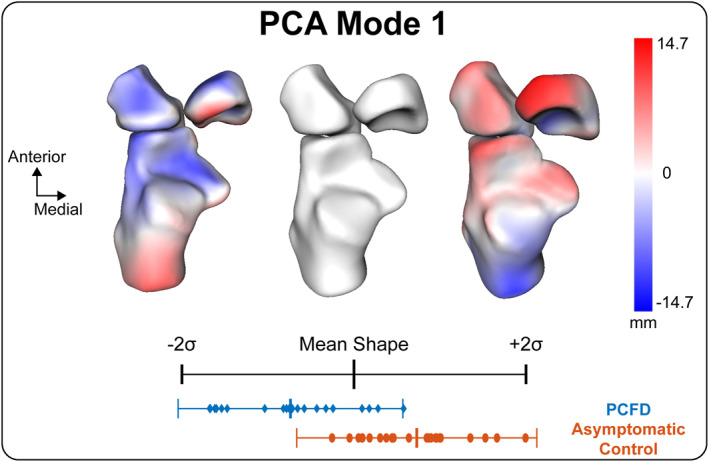
Multi‐domain PCA mode 1 quantitative visual description focused on talonavicular joint from the superior viewing perspective. The red regions indicate outward displacement, and the blue regions indicate inward displacement from the mean shape, measured along the surface normal at each point.

The information regarding the subjects' characteristics can be found in Table 1. Six PCA modes were retained for analysis and described 85.4% of the overall shape variation. The individual six modes (1–6) contained 53.0%, 15.8%, 5.6%, 5.1%, 3.2%, and 2.7% of the explained variation, respectively (Table 2). Anatomical variations were observed across each of these modes. Distinct patterns of coordinated morphological variation were identified among joints, together with changes in 3D foot alignment. Comparing the mean shape parameters of PCFD and asymptomatic controls for these modes, the first mode of variation in multi‐domain SSM was the only mode with significant differences in PCA component scores (Table 2). According to the variation in PCA scores between PCFD and asymptomatic controls, the −2 SD shape represented an extreme variation toward PCFD morphology (Figures 2, 3). In PCFD, the anterior and medial surfaces of the subtalar joint tended to separate when the talus was plantarflexed and internally rotated, while the calcaneus showed plantarflexion and inversion. Similarly, the navicular, cuboid, and cuneiform bones shifted medially and downward with internal rotation. The Chopart joint demonstrated morphological changes that could be qualitatively interpreted as a tendency toward dorsiflexion, external rotation, and inversion (Figure 2). In contrast, the Lisfranc joint was abducted and dorsiflexed while everted, which was opposite to the subtalar and Chopart joint patterns (Figure 2). The metatarsals, particularly the second metatarsal, was displaced posteriorly by this deformity. In addition, the talonavicular joint exhibited fan‐shaped medial gapping, which was clearly observed in patients with PCFD (Figure 3). Three‐dimensional subtalar morphology is further demonstrated in Videos [Link], [Link], [Link].

**TABLE 1:** Participant demographics.

	Case (*n*)	Age (years)	Height (cm)	Weight (kg)	BMI (kg/m^2^)	Meary's angle (°)	Calcaneal pitch (°)	Hindfoot alignment angle (°)
Asymptomatic control	23	47.8 ± 19.2	155.0 ± 7.2	58.1 ± 14.1	23.9 ± 5.5	−10.6 ± 9.1	17.7 ± 5.8	1.4 ± 6.8
PCFD	23	65.1 ± 7.1	154.0 ± 4.9	60.4 ± 9.8	25.1 ± 4.3	−36.2 ± 12.7	10.1 ± 8.1	16.4 ± 11.4
*p*‐value (*α* = 0.05)		**< 0.01**	> 0.05	> 0.05	> 0.05	**< 0.01**	**< 0.01**	**< 0.01**

*Note:* Bolded values indicate significant differences between groups.

**TABLE 2:** Retained modes of variation.

	Mode 1	Mode 2	Mode 3	Mode 4	Mode 5	Mode 6
Explained variance (eigenvalue)	53.0%	18.8%	5.6%	5.1%	3.2%	2.7%
*p*‐value (*α* = 0.05)	**4.16E‐9**	0.49	0.66	0.97	0.91	0.81

*Note:* Bolded values indicate significant differences between groups.

## 4 Discussion

This study used a multi‐domain SSM to analyze and quantify 3D foot and ankle alignment and shape variation from the tibia to the metatarsals in females presenting with PCFD compared to their asymptomatic control counterparts. The relevant findings from the multi‐domain SSM suggest that the deformity in multi‐joint alignment is in the same direction from the subtalar joint to the Chopart joint with presentation of dorsiflexion, abduction, and inversion. Meanwhile, the Lisfranc joint undergoes frontal plane eversion alignment shifts to compensate for peritalar subluxation, while still being dorsiflexed and abducted. Our results demonstrated that the subtalar and Chopart joints showed coordinated morphological changes, whereas the Lisfranc joint exhibited variation in the opposite direction. These findings highlight a distinct compensatory relationship among midfoot joints in PCFD. Although quantitative deviations at each joint were not directly measured, the strength of this study lies in the multi‐domain approach, which enabled simultaneous assessment of all foot joints and highlighted their compensatory relationships in PCFD.

In some cases of osteoarthritis of the ankle, a so‐called compensatory functional relationship in the coronal plane of the hindfoot has been reported between the ankle joint and the subtalar joint [14]. A similar phenomenon occurs in the PCFD, suggesting a compensatory relationship between the Chopart and Lisfranc joints in the coronal plane of the midfoot. In addition, Miyamoto et al. have shown that the kinematics of the subtalar and talonavicular joints are strongly correlated and exhibit similar motion patterns, as well as comparable compensatory relationships with the ankle joint kinematics [21]. The presentation of deformity as well as joint kinematics are likely to be linked to the subtalar and talonavicular joints, and as a result, the subtalar and Chopart joints are strongly correlated in the deformity, and it may be the origin of the deformation of the PCFD. These findings have potential clinical implications. The identification of coordinated changes in the subtalar and Chopart joints, along with variation in the opposite direction in the Lisfranc joint, may help surgeons anticipate how correction of hindfoot alignment could influence midfoot and forefoot posture. Such insights may assist in surgical planning to avoid overcorrection or undercorrection and could also provide prognostic information about the progression of deformity in patients with PCFD.

The effect of instability of the talonavicular joint due to injury of the spring ligament on the presentation of PCFD deformity has often been discussed [22, 23]. However, it is not yet clear how alignment changes in the talonavicular joint develop in PCFD. Mode 1 results showed a statistically significant difference between PCFD and asymptomatic controls, where there was a talonavicular joint gap medially in a fan shape in the PCFD group. This may have been caused by tearing or elongation of the spring ligament present at the joint. Although no significant difference was observed, the medial gapping of the talonavicular joint was also observed in modes 2–4. Therefore, medial gapping of the talonavicular joint may be a critical clinical factor in the presentation of PCFD.

Our results also showed the metatarsals were displaced posteriorly in the PCFD group and that displacement was particularly large in the second metatarsal. There has been frequent discussion regarding the association between metatarsal length and flatfoot [24, 25]. Morton et al. [24] reported that a short first metatarsal bone causes collapse of the longitudinal arch, whereas Robert et al. [25] reported no relationship between first metatarsal bone length and flatfoot. In our previous study [26], we also found no difference in each metatarsal length between PCFD and asymptomatic controls. This suggests that the apparent metatarsal length changes in PCFD may not reflect alterations in actual bone length, which can be directly measured from CT, but rather relative length differences due to alignment changes, resulting in a functionally shorter second metatarsal than the others.

This study compared PCFD and asymptomatic groups with a focus on females. PCFD occurs three times more often in women [5], and bone morphology of the foot and ankle is considered to differ between men and women [6]. However, most studies of PCFD morphology have evaluated both sexes together. In addition, our previous study showed that patients with PCFD exhibited 3D differences in the shape of the distal fibula, talus, calcaneus, navicular, and cuboid [26]. This means that evaluating males and females together may confound alignment findings because of sex‐related differences in bone morphology. As a result, this study highlights that variations in hindfoot bone morphology may influence overall foot alignment.

The PCFD classification developed in 2020 has been more widely accepted and used in clinical practice and the accuracy of clinical use has been tested and verified [4, 27, 28]. In this recent classification, the “Class” is divided according to the presence or absence of deformity of the hindfoot valgus, midfoot abduction, forefoot varus, peritalar subluxation, and ankle instability. This classification breakdown suggests that PCFD is not necessarily accompanied by deformity present at the Lisfranc joint or subluxation of the subtalar joint. Our multi‐domain SSM results demonstrated subtalar joint subluxation and Lisfranc joint deformity. This may have been influenced by the fact that more than half of our cases were severe cases with a PCFD classification of 2C or higher. It has been suggested that the stage classification changes depending on the presence or absence of compensatory function of the subtalar joint in osteoarthritis of the ankle [14]. Similarly, compensatory function of the Lisfranc joint differences may have a very strong influence on the classifications and is not likely to occur in all PCFD patients. Therefore, future analysis should focus on class presentations, accompanied by a substantially larger population for modeling inputs.

Our study has limitations. First, our study does not allow evaluation of causality. The observed group differences in 3D foot alignment may therefore reflect either consequences or contributors to PCFD or a combination of both. To clarify this, further research, including longitudinal studies, is necessary. Second, there was an absence of age matching between individuals with PCFD and asymptomatic participants. This is because asymptomatic controls were selected without foot deformity or trauma, and many of the subjects were younger. An age‐matched study design should be considered in the future. Third, this study focused on only females. This allowed us to focus on the differences in PCFD without gender differences, but we do not know whether similar results occur in males, and further investigation of PCFD in males is needed. Fourth, this study did not examine the structures of ligaments, tendons, and articular cartilage. Fifth, the CT used in this study is simulated weight‐bearing and not fully loaded. However, a recent study determined that partial weight‐bearing greater than 25% bodyweight were sufficient to reproduce full weight‐bearing sagittal foot alignments [29]. Therefore, these factors may have influenced the results to a varying extent. Sixth, although SSM visualizes 3D morphological differences beyond simple 2D measures, it cannot provide direct quantitative joint angles in local coordinate systems. Thus, our interpretations remain qualitative, and future studies should integrate SSM with coordinate‐based analyses for clinically meaningful quantification. Seventh, as DISIOR provides surface‐based reconstructions rather than voxel‐level segmentations, some fine morphological details may not have been fully captured. Finally, the smaller sample size represents a limitation of this study. Statistical shape modeling generally requires larger cohorts to achieve robustness and fully represent population‐level variation, and therefore, our findings should be interpreted with caution. Because of this limited cohort size, mirroring was used to increase the number of surface models during SSM construction. Although mirroring is an accepted preprocessing step in prior SSM studies, it does not add pathology‐specific variation and may influence population‐level variability.

## 5 Conclusion

This study demonstrated distinct morphological differences between females with PCFD and asymptomatic controls, including coordinated changes in the subtalar and Chopart joints, variation in the opposite direction in the Lisfranc joint, and medial fan‐shaped gapping of the talonavicular joint. These findings suggest that hindfoot and midfoot deformities are interrelated, with potential implications for surgical planning and prognosis. Future research, including longitudinal studies and sex‐specific comparisons, is warranted to clarify deformity progression and refine clinical grading of PCFD.

## Author Contributions

**Takuma Miyamoto:** conceptualization, data curation, formal analysis, funding acquisition, investigation, visualization, writing – original draft, writing – review and editing. **Rich J. Lisonbee:** formal analysis, supervision, validation, writing – review and editing. **Kassidy Knutson:** formal analysis, writing – review and editing. **Hiroaki Kurokawa:** data curation, writing – review and editing. **Akira Taniguchi:** data curation, writing – review and editing. **Yasuhito Tanaka:** data curation, investigation, writing – review and editing. **Amy L. Lenz:** conceptualization, methodology, project administration, resources, software, writing – review and editing.

## Funding

This work was supported by Nakatani Foundation International Fellowship (2022H1002) and JSSF International Fellowship Foundation. The funding sources were not involved in study design; in the collection, analysis, and interpretation of data; in the writing of the report; and in the decision to submit the article for publication. Funding provided by the National Institutes of Health supported Dr. Lenz (NIH K01AR080221), and ShapeWorks software (U24EB029011, R01AR076120).

## Ethics Statement

This study was conducted in accordance with ethical standards and was approved by the Institutional Review Board (IRB) (No 2872). An opt‐out statement regarding the application of medical data was published on our institute's website. This study was performed under the principles of the World Medical Association Declarations of Helsinki. No material from other sources was reproduced, and this study was not a registered clinical trial.

## Conflicts of Interest

The authors declare no conflicts of interest.

## Supporting information


**Video S1:** Video representation of the PCFD mean shape to control mean shape animation of whole foot alignment and morphology in the axial plane.

**Video S2:** Video representation of the PCFD mean shape to control mean shape animation of whole foot alignment and morphology in the sagittal plane.

**Video S3:** Video representation of the PCFD mean shape to control mean shape animation of whole foot alignment and morphology in the coronal plane with forefoot hidden to visualize the cuboid, navicular, and hindfoot anatomy.

## Data Availability

The data that support the findings of this study are available from the corresponding author upon reasonable request.
